# Indication of Liver Transplantation for Hepatocellular Carcinoma Should Be Reconsidered in Case of Microvascular Invasion and Multilocular Tumor Occurrence

**DOI:** 10.3390/jcm10061155

**Published:** 2021-03-10

**Authors:** Jan-Paul Gundlach, Stephan Schmidt, Alexander Bernsmeier, Rainer Günther, Victor Kataev, Jens Trentmann, Jost Philipp Schäfer, Christoph Röcken, Thomas Becker, Felix Braun

**Affiliations:** 1 Department of General, Visceral-, Thoracic-, Transplantation- and Pediatric Surgery, Campus Kiel, University Medical Center Schleswig-Holstein (UKSH), Arnold-Heller-Strasse 3, 24105 Kiel, Germany; stephanschmidt90@googlemail.com (S.S.); Alexander.Bernsmeier@uksh.de (A.B.); Thomas.Becker@uksh.de (T.B.); Felix.Braun@uksh.de (F.B.); 2 Department of Internal Medicine I, UKSH, Campus Kiel, Arnold-Heller-Strasse 3, 24105 Kiel, Germany; Rainer.Guenther@uksh.de (R.G.); Victor.Kataev@uksh.de (V.K.); 3 Institute of Radiology and Neuroradiology, UKSH, Campus Kiel, Arnold-Heller-Strasse 3, 24105 Kiel, Germany; Jens.Trentmann@uksh.de (J.T.); JostPhilipp.Schaefer@uksh.de (J.P.S.); 4 Department of Pathology, UKSH, Campus Kiel, Arnold-Heller-Strasse 3, 24105 Kiel, Germany; Christoph.Roecken@uksh.de

**Keywords:** HCC, hepatocellular carcinoma, liver transplantation, microvascular invasion, multilocular, downstaging, bridging, TACE

## Abstract

Liver transplantation (LT) is routinely performed for hepatocellular carcinoma (HCC) in cirrhosis without major vascular invasion. Although the adverse influence of microvascular invasion is recognized, its occurrence does not contraindicate LT. We retrospectively analyzed in our LT cohort the significance of microvascular invasion on survival and demonstrate bridging procedures. At our hospital, 346 patients were diagnosed with HCC, 171 patients were evaluated for LT, and 153 were listed at Eurotransplant during a period of 11 years. Among these, 112 patients received LT and were included in this study. Overall survival after 1, 3 and 5 years was 86.3%, 73.9%, and 67.9%, respectively. Microvascular invasion led to significantly reduced overall (*p* = 0.030) and disease-free survival (*p* = 0.002). Five-year disease-free survival with microvascular invasion was 10.5%. Multilocular tumor occurrence with simultaneous microvascular invasion revealed the worst prognosis. In our LT cohort, predominant bridging treatment was transarterial chemoembolization (TACE) and the number of TACE significantly correlated with poorer overall survival after LT (*p* = 0.028), which was confirmed in multiple Cox regression analysis for overall and disease-free survival (*p* = 0.015 and *p* = 0.011). Microvascular tumor invasion is significantly associated with reduced prognosis after LT, which is aggravated by simultaneous occurrence of multiple lesions. Therefore, indication strategies for LT should be reconsidered.

## 1. Introduction

The incidence of hepatocellular carcinoma (HCC) is increasing in Western countries [1]. HCC regularly develops as a result of chronic liver disease [2,3]. Therapeutic options consist of resection, locoregional procedures such as radiofrequency ablation (RFA), and transarterial chemoembolization (TACE) as well as liver transplantation (LT) and immunomodulatory therapies. LT is standard therapeutic treatment in patients with underlying cirrhosis (around 80% of all HCC cases [3]) and tumor manifestation within Milan criteria (one node < 5 cm or up to three nodules < 3 cm without major vascular invasion) [2]. LT beyond Milan criteria impairs outcome due to vascular invasion and number of nodules [4,5]. However, the University of California, San Francisco score (UCSF score; one node ≤ 6.5 cm or a maximum of three lesions ≤ 4.5 cm with all lesions ≤ 8 cm without major vascular invasion) has been proven to predict equivalent survival rates [6]. Interestingly, while the microvascular invasion is well known for its implication on tumor survival [2,5,7,8], its presence has not been merged into outcome predictions in different classification systems, so far.

Due to the increasing demand for donor organs and the moderate willingness to donate organs, the best possible therapy planning for HCC and the selection of patients for LT as well as the optimization of bridging procedures remain important tasks. Locoregional procedures, especially TACE [9], are well-known for its downstaging properties as well as bridging characteristics until an organ is available [10]. In a recent review [11], downstaging methods were reported to be successful irrespective of the method in about every second patient treated. However, within the quoted reports, the inclusion and response criteria were not standardized. Thus, recurrence rates after LT are higher in the downstaged HCC cohort compared to patients being initially treated within transplantation criteria and differ widely: 5-year survival after LT in downstaged patients ranged from 54.6% to 94.1% [11]. However, response to TACE is a good predictor for outcome after transplantation [12,13]. Maximum tumor number and size should be limited for downstaging. The UCSF protocol recommends downstaging for HCC ≤ 8 cm for single as well as for multiple tumors (two to three tumors with each tumor ≤ 5 cm or four to five tumors with each tumor ≤ 3 cm) [14]. Treatment success should be rated by the generally accepted radiologic downstaging classification, the mRECIST (modified Response Evaluation Criteria in Solid Tumors) score [15].

Although, as highlighted above, not only the maximum tumor size and number of tumor nodes are relevant for the outcome, the microvascular invasion is recognized to be important for overall and disease-free survival. Therefore, we intended to clarify the importance of microvascular invasion as well as the number of tumor lesions against the background of organ shortage.

## 2. Materials and Methods

The present study includes patients with an admission diagnosis of HCC who presented at our transplant unit of the University Medical Center Schleswig-Holstein, Campus Kiel from January 2006 to December 2016. The data was collected after approval by our local ethics committee (AZ: D 566/15). The diagnosis is based on the international statistical classification of diseases and related health problems (ICD-10-GM) with the diagnosis code C22.0 (hepatocellular carcinoma). A patient cohort of 403 patients was created. This cohort includes patients whose initial diagnosis was made at our hospital but also patients who were referred from other hospitals for further treatment. Furthermore, patients were initially included who wanted to be treated close to home after diagnosis.

Documented causes of liver damage were: autoimmune hepatitis (AIH), primary sclerosing cholangitis (PSC), alcohol-toxic liver damage, non-alcoholic fatty liver hepatitis (NASH), alpha-1-antitrypsin deficiency, hemochromatosis and infection by the hepatitis viruses A, B, C, D, and E.

Non-HCC patients were excluded from the study. The size of the largest tumor, the total number of all lesions, the size of the individual tumors (>8 cm, 5–8 cm, 3–5 cm, 2–3 cm, <2 cm), and whether there was vascular invasion and extrahepatic metastases were documented. The chances of success before LT were assessed according to the Milan criteria and the UCSF score. As highlighted above, both scores exclude extrahepatic metastases and major vascular invasion.

Date of first listing on the Eurotransplant waiting list was documented. Dropouts from the waiting list occurred due to tumor progression, incompliance, or death. Bridging was performed by means of locoregional procedures (such as resection, TACE, selective internal radiotherapy (SIRT), RFA, or percutaneous ethanol injection (PEI)) during the waiting period. In the event of delisting, it was also recorded whether the patients were under ongoing locoregional therapy.

The date of the last follow-up and possible tumor recurrence as well as tumor-related or independent death during the study were recorded. LT procedures were published before [16]. All explants were examined macroscopically and histologically by board certified surgical pathologists. HCCs were classified based on histological criteria of the World Health Organization and the TNM-classification of the UICC. The survival time analyses were carried out using the Kaplan–Meier method. Simple and multiple Cox regression were used to determine the various factors possibly influencing overall survival and disease-free survival such as tumor classification, diagnosis of liver disease, model for end-stage liver disease (MELD) score, Child-Pugh score, Milan/UCSF stages, UICC staging, singular und multiple tumor occurrence, alpha-fetoprotein (AFP)-levels as well as bridging treatment methods such as TACE, PEI, resection, and RFA. A *p*-value ≤ 0.05 was determined to be statistically significant. Hazard ratio (HR) and 95% confidence interval (CI) are stated.

## 3. Results

### 3.1. Baseline Clinical Presentation

**Figure 1:**
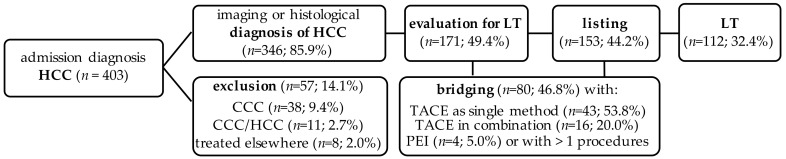
Flowchart of the LT cohort. Bridging with >1 procedures included: PEI, radiofrequency ablation, TACE and/or resection. Abbr.: CCC: cholangiocellular carcinoma; HCC: hepatocellular carcinoma; LT: liver transplantation; PEI: percutaneous ethanol injection; TACE: transarterial chemoembolization.

In the present study, a cohort of 403 patients was examined (Figure 1), who were suspected of having a malignant liver tumor diagnosed between January 2006 and December 2016. There were 291 (72.2%) male and 112 (27.8%) female patients. The mean age at first diagnosis was 67.9 ± 12.3 years, the youngest patient was 7 years, and the oldest patient was 101 years. Of the 403 patients, 346 patients received histologically and/or image-morphologically HCC diagnosis (85.9%). In 38 patients, a cholangiocellular carcinoma (CCC) (9.4%) was found histologically, in 11 patients (2.7%) a mixed tumor of an HCC and a CCC was present, and 8 patients (2%) remained without histological evidence of a malignant tumor. This resulted in a corrected total cohort of *n* = 346 patients. The mean age at first diagnosis in this corrected cohort was 68.4 ± 12.1 years, the youngest patient was diagnosed with HCC at 17 years of age, and the oldest patient was 89 years old at the time of first diagnosis; 266 patients (76.9%) were male and 80 (23.1%) were female. The average BMI of the patients was 26.7 ± 5.0 kg/m^2^.

Liver cirrhosis was found in 273 patients (78.9%) of the HCC cohort; 131 patients (37.9%) suffered of viral hepatitis, and 121 (35.0%) had an alcohol-related cirrhosis of the liver. In the LT group, most of the patients (95.5%) had cirrhosis as the underlying disease, and 49.1% suffered from hepatitis, most commonly being an infection with hepatitis C. The exact distribution of the underlying diseases can be found in Table 1.

Table 2 presents the HCC data. At the time of initial HCC diagnosis, 204 patients (59.0%) showed a solitary tumor, and 142 patients (41.0%) had multilocular tumor involvement. Extrahepatic metastases were found in 28 patients (8.1%). An increased AFP value was initially detected in 227 patients (65.6%). In the subgroup of patients with LT, a solitary HCC lesion was most common (*n* = 74; 66.1%). Fourteen patients were outside Milan and UCSF-in: 88 patients (78.6%) did not exceed the UCSF criteria. Thereof, 74 patients (66.1%) were within the Milan criteria at the time of initial diagnosis. A histological diagnosis was made in 40 cases (35.7%) before LT by prior resection (*n* = 24, 21.4%) and/or biopsy (*n* = 22, 19.6%). Biopsy was intended to rule out CCC or HCC/CCC tumors.

### 3.2. Bridging and Downstaging before Liver Transplantation

Bridging before LT was performed in 45 patients (60.8%) of the total of 74 patients who met the Milan criteria at the time of initial diagnosis. Most of these patients (27; 60.0%) were treated with TACE. Four patients (8.9%) underwent PEI and 14 (31.1%) underwent several procedures (TACE and PEI, resection and RFA, TACE and resection) prior to LT. Within this group, 11 patients received combined procedures including TACE. Fourteen patients received treatment before listing at Eurotransplant: Resection and TACE were performed in 7 patients (6.3%) each.

In the group of transplant patients, who did not meet the Milan criteria at initial diagnosis (*n* = 36), most patients were treated with TACE (16; 45.7%). In 19 patients (52.8%) several procedures were used for downstaging before LT. Within this group, 5 patients received combined procedures including TACE. One patient outside Milan received a living donor LT (LDLT).

The remaining 17 patients (15.2%) received no therapy before transplantation. In two of them, HCC was diagnosed after histological examination; at the time of LT, they were formally outside Milan and UCSF.

A waiting list dropout occurred in 34 patients (22.2%) of the 153 patients during the study period, and tumor progression occurred in 18 patients (52.9%), including 16 patients (47.1%) who received progression under TACE therapy. Two patients (5.9%) were removed from the waiting list because of tumor-free resected tissue. Four patients (11.8%) had to be removed from the list due to non-compliance. Noteworthy, 10 patients (29.4%) died during the waiting period.

### 3.3. Liver Transplantation and Outcome

One hundred seventy-one (49.4%) of 346 patients with HCC were evaluated for LT, 153 patients (44.2%) were listed at Eurotransplant, and 112 (32.4%) received an LT during the study period. Most commonly, full-size organs from deceased donors (DDLT) were transplanted (*n* = 90; 80.4%), 9 patients (8.0%) received a split organ, and 3 patients (2.7%) received a domino transplantation. The mean waiting time for a donor organ was 8.68 ± 11.17 months. Ten patients (8.9%) received a LDLT. UICC tumor stages pT1, pT2, pT3a, pT3b, and pT4 were found in 46, 39, 5, 8, and 1 of the cases after LT, respectively (46.5%, 39.0%, 4.5%, 7.1%, and 0.9%). In 12 cases, the pathologists did not find any tumor left after neoadjuvant treatment. In 2 cases (1.8%), positive lymph nodes were found. Tumor grading was missing in 35 cases (31.3%) after locoregional therapy. In other tumors, differentiation was found for G1, G2, and G3 in 18 (16.1%), 53 (47.3%), and 6 cases (5.4%), respectively.

**Figure 2:**
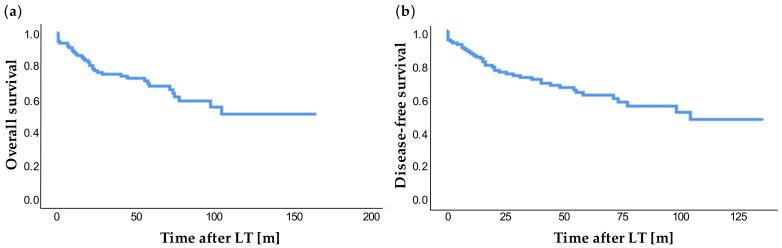
Overall survival (**a**) and disease-free survival (**b**) after LT. Time in years [y].

In 14 patients (12.5%) with LT, a tumor recurrence was diagnosed during the observation period. This occurred on average after 28.16 ± 26.19 months (range: 2.2 months to 98.4 months). At the study end point, 7 patients were waiting for an organ. Overall survival (Figure 2a) showed 1-, 3-, and 5-year survival rates of 86.3%, 73.9%, and 59.6%, respectively. The 1-, 3-, and 5-year disease-free survival was 84.5%, 71.1%, and 59.6%, respectively (Figure 2b).

### 3.4. Factors Associated with Impaired Outcome: Microvascular Invasion, Multilocular HCC and Number of TACE Treatments

**Figure 3:**
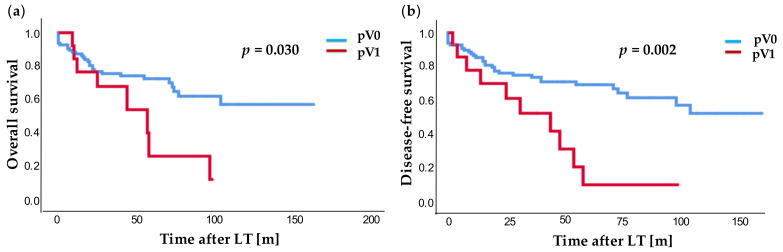
Overall survival (**a**) and disease-free survival (**b**) after LT with (red) and without (blue) microvascular infiltration. Time in years [y].

We investigated whether the type of underlying liver disease in the LT cohort had an influence on survival and disease-free survival after transplantation. We could not find any statistical significance for alcoholic cirrhosis (*p* = 0.106; *p* = 0.248), NASH cirrhosis (*p* = 0.356; *p* = 0.981) and hepatitis (*p* = 0.502; *p* = 0.456). Next, microvascular invasion of the tumor was examined (Figure 3). In our cohort, we found a significantly poorer overall survival (*p* = 0.030) in patients with microvascular invasion (pV1) compared to patients without microvascular invasion (pV0, Figure 3a): 1-, 3-, and 5-year survival rates were for pV0 vs. pV1 86.4% vs. 76.9%; 74.6% vs. 54.7%; and 70.5% vs. 13.7%, respectively. Patients with microvascular invasion showed a highly significantly poorer disease-free survival (*p* = 0.002, Figure 3b). The 1-, 3-, and 5-year disease-free survival was 85.4% vs. 70.1%; 73.8% vs. 42.1%; and 67.0% vs. 10.5%, respectively. Intriguingly, among the patients with microvascular invasion of HCC, about 90% suffered tumor relapse after 5 years.

**Figure 4:**
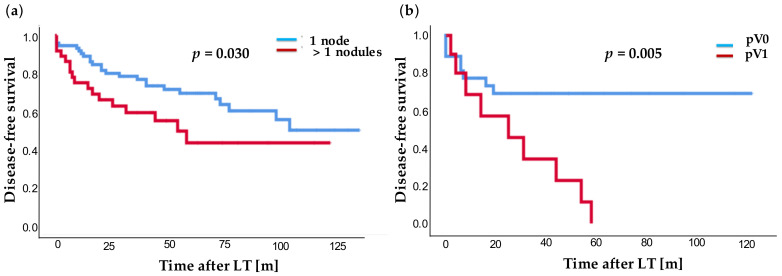
(**a**) Disease-free survival after LT for single (blue) vs. multilocular (red) lesions after LT and (**b**) disease-free survival for patients after LT with multilocular lesions and with microvascular infiltration (red) or without microvascular infiltration (blue). Time in years [y].

In addition, overall and disease-free survival of patients with solitary HCC lesion and patients with more than one tumor lesion in the explanted organ were examined. Overall survival (*p* = 0.050) as well as disease-free survival (*p* = 0.030, Figure 4a) were significantly better for patients with a solitary lesion in contrast to patients with multilocular lesions: 1-, 3-, and 5-year survival rates for single vs. multilocular lesions of 90.4% vs. 74.9%; 78.0% vs. 60.8%; and 70.8% vs. 54.1% as well as disease-free survival of 89.1% vs. 72.1%; 76.9 vs. 55.0%; and 66.6% vs. 43.3%. Noteworthy, the disease-free survival after LT was highly significantly worse (*p* = 0.005, Figure 4b) for patients with multilocular HCC lesions and additional microvascular invasion (pV1) compared to patients without additional microvascular invasion (pV0).

In simple Cox-regression analysis, the presence of microvascular invasion (pV1: *p* = 0.039, HR: 2.299; CI: 1.045–5.062), multilocular lesions (*p* = 0.001; HR: 1132; CI 1.052–1.219), or more than one treatment with TACE before LT (number of TACE, *p* = 0.028, HR: 1.131; CI: 1.014–1.262) were revealed to have a significantly poorer impact on overall survival. In multiple Cox-regression analysis, the number of HCC lesions (*p* = 0.002; HR: 1.133; CI: 1.049–1.224) and the total number of TACE (*p* = 0.015; HR: 1.178; CI: 1.032–1.344) showed a highly significant negative impact on overall survival after LT. In multiple Cox-regression, the disease-free survival was demonstrated to be significantly influenced by microvascular invasion (*p* = 0.039; HR: 2.374; CI: 1.045–5.395); number of tumor lesions (*p* = 0.008; HR: 1.114; CI: 1.029–1.206) and number of TACE (*p* = 0.011; HR: 1.159; CI: 1.035–1.298). In addition, the simple Cox regression showed a significantly negative correlation for the pT2 stage and the MELD score with overall and disease-free survival. Diagnosis of a multilocular HCC before LT and the total number of lesions diagnosed before LT showed in simple Cox regression analysis significantly reduced disease-free survival. In multiple Cox regression, the last MELD score before LT was found to have a highly significant correlation with reduced overall survival (*p* < 0.001; HR: 1.067; 1.013–1.104), while the highest MELD score during the waiting list period showed a highly significant correlation with worsened disease-free survival (*p* < 0.001; HR: 1.060; CI: 1.026–1.095).

## 4. Discussion

Liver cirrhosis is known to be the most important risk factor for the development of HCC. In our study, 78.9% of the entire HCC cohort and 95.5% of the patients with LT had a cirrhosis. Chronic hepatitis B is the most common underlying disease for the development of cirrhosis and is responsible for around 50% of the cases worldwide due to deficient vaccination in Asia and sub-Saharan Africa [1]. To the contrary, hepatitis B was found in only 16.5% of our patients and hepatitis C in 23.7%. Alcohol induced cirrhosis was the most common cause of liver cirrhosis in our cohort, i.e., 35.0%, resembling average results for Western European countries [17]. In recent years, NASH has also become increasingly important in the Western world as a cause for liver cirrhosis. It was the third most common cause of cirrhosis in our series (7.8%).

Due to organ shortage, risk stratification of disease-free survival after LT is of great relevance for HCC treatment and forms the basis for a lively debate. Accordingly, a recently proposed dropout calculation from patients on the waiting list comprises the Child-Pugh score, number of tumor lesions, AFP value, and the MELD score [18]. However, decision support is needed for the transplantation criteria of patients beyond Milan/UCSF limits.

### 4.1. Microvascular Invasion & Multilocular Tumor Occurrence

At present, microvascular invasion after resection is not a contraindication for LT. Nevertheless, outcome analysis showed that patients with microvascular invasion (pV1) had a significantly poorer overall survival (*p* = 0.030) and disease-free survival (*p* = 0.002) compared to patients without microvascular invasion (pV0). In international studies, the microvascular invasion has been proven several times to be an important prognostic factor for overall survival and disease-free survival after LT in HCC [5,19,20,21]. In line, resected tumors with identification of microvascular invasion are recommended to be not considered early-stage tumors with regard to Barcelona Clinic Liver Cancer (BCLC) classification [22]. Identification of microvascular infiltration is crucial for a proper classification. Different models have been proposed for the prediction of microvascular invasion [23,24,25,26,27,28]: Attractive seems the neutrophil-lymphocyte ratio (NLR), which was demonstrated and validated as an independent risk factor for microvascular invasion [27,28]. Nevertheless, the data has not been validated in a prospective setting so far. For MR imaging, we recommend rather endothelial permeable contrast agents instead of hepatocyte-specific contrast media as a possibility to improve diagnosis.

Multilocular tumor occurrence was associated with a significantly shorter overall survival (*p* = 0.050, 5-year survival rate: single lesion 70.8% vs. multilocular HCC 54.1%) and disease-free survival (*p* = 0.030, 5-year survival rate: single lesion 66.6% vs. multilocular HCC 43.3%) in our study, as demonstrated by Kaplan–Meier analysis. This is in contrast to other groups, who demonstrated the size of the major lesion to be more important than the number of tumor lesions [29], or the total tumor volume to be most important for survival prediction [30].

Yet, in combination with microvascular invasion, multilocular tumor occurrence had a highly significant negative influence on the disease-free survival (*p* = 0.005). In simple and multiple Cox regression analysis, the number of histologically detected HCC lesions in the explant could be identified as a negative influencing factor for both overall and disease-free survival (*p* = 0.001, HR: 1.132). In the multiple Cox regression analysis, the number of histological HCC lesions was confirmed as an independent risk factor (*p* = 0.002; HR: 1.133). Simple Cox regression showed no difference in overall survival between patients with solitary or multilocular HCC manifestation.

### 4.2. Number of Performed TACE Procedures Is Associated with Reduced Overall Survival

Response to TACE treatment is well known to be a possible selection criterion for LT candidates [12,18]. It was shown that low-grade tumors responded better than high-grade tumors (G0 to G4) with more TACE procedures being required in higher graded tumors [31], although no systematic HCC grading exists [32]. In our study, out of a total of 346 HCC patients, 182 patients (52.6%) received TACE treatment. In the subgroup of patients initially outside Milan, a total of 35 patients were successfully downstaged and subsequently transplanted within Milan criteria. In general, single and combination therapeutic regimen with TACE were used in 44.6% and 14.3% of the LT cohort, respectively. Simple Cox regression was used to demonstrate that patients with HCC after LT had a significantly poorer overall survival the more often TACE was performed (*p* = 0.028); the number of TACEs was also confirmed in the multiple Cox regression as a significant independent negative factor for overall and disease-free survival (*p* = 0.015 and *p* = 0.011, respectively). This phenomenon has been addressed before: different instruments for decision making of TACE re-treatment were introduced. The ART and the ABCR score were recently developed [33,34]. Both scores include changes in the Child-Pugh score as well as radiological tumor response to the first TACE treatment. The ART score includes serum AST increase after the first TACE, whereas the ABCR score additionally includes AFP level at baseline as well as the BCLC stage at baseline. Nevertheless, both scores have not been implemented in clinical routine due to insufficient predictive value so far [9].

### 4.3. Tumor Biopsy for Detection of Microvascular Infiltration and Poorly Differentiated Tumors

Tumor biopsies are not performed on a regular basis due to the risk of needle track seeding—which is described in a recent review with an incidence of 3% to 9% and, most of all, due to the accurate radiological diagnosis following the LIRADS score (introduced by the American College of Radiology *Liver Imaging Reporting and Data System*) [35]. In our cohort, 19.6% of the patients received a biopsy prior to the LT. Our cohort includes patients who are diagnosed according to the rules of the German Medical Association based on histological or radiological criteria. At present, we use the LIRADS classification at our institution. In LIRADS 4 cases, or unclear cases (non-LIRADS 5 but supposed HCC), we perform biopsy for diagnosis of HCC in order to rule out CCC or HCC/CCC. Tumors between 1–2 cm are diagnosed by two contrast enhanced imaging procedures, while tumors >2 cm require only 1 contrast-enhanced imaging method according to the EASL guideline [17]. Whenever possible, imaging is preferred over biopsy to avoid needle track seeding. Nevertheless, in case of tumors beyond Milan criteria, biopsy is recommended for the biggest tumor node in order to exclude poorly differentiated HCC from LT [36]. In a prospective validation study, the extended Toronto Criteria were validated in 243 patients: 43% of the patients exceeding Milan criteria received biopsy. Patients without poorly differentiated tumors were transplanted with comparable results with patients transplanted within Milan criteria [37], although difficulties of tumor grading remain [32]. As highlighted above, tumor grading influences response to bridging treatment [31]. We therefore propose the discussion of tumor biopsy for detection of microvascular invasion and poorly differentiated tumors, with special emphasis on the worsened prognosis in case of multilocular tumor occurrence with simultaneous microvascular invasion. In particular, biopsy of more than one node for estimation of survival after LT should be discussed.

This study has several limitations. First of all, since this study is a retrospective analysis, its validity is limited with regard to the parameters collected. In particular, a detailed listing of the histological results prior to LT would be of interest for the specification of microvascular infiltration. In addition, our data also suggests that future studies should investigate sample errors in multiple lesions. Since microvascular invasion is not a contraindication for LT in Germany, the impact of preoperative diagnosis should be focused. Furthermore, the justification for salvage LT after preceding resection should be discussed further in the case of histological evidence of pV1 status in the resected specimen.

## 5. Conclusions

LT is considered a standard of care treatment option in HCC in cirrhosis. Despite accurate tumor diagnosis via means of radiographic methods, our results emphasize the need of precise identification of microvascular invasion. Simultaneously, multilocular occurrence is recognized as an important prognostic marker for overall and disease-free survival after LT in HCC. Decision support is needed for the transplantation criteria of patients beyond Milan/UCSF limits. Prospective validation of microvascular invasion prediction (by radiological means) as well as analysis of microvascular invasion-associated underlying risk-factors should be tackled. Locoregional treatment with TACE is well-known for its downstaging abilities; nevertheless, the reasons for a possibly reduced outcome after LT following repeated TACE interventions should be focused in further studies.

## Figures and Tables

**Table 1:** Underlying disease of liver degeneration in the entire HCC and the LT cohort.

Diagnosis	HCC Cohort (*n* = 346) [*n* (%)]	Patients with LT (*n* = 112) [*n* (%)]
**cirrhosis**	273 (78.9)	107 (95.5)
**autoimmune hepatitis**	6 (1.7)	3 (2.7)
**α1-antitrypsin deficiency**	2 (0.6)	2 (1.8)
**hemochromatosis**	2 (0.6)	1 (0.9)
**alcoholic cirrhosis**	121 (35.0)	41 (36.6)
**NASH**	27 (7.8)	10 (8.9)
**hepatitis**	131 (37.9)	55 (49.1)
**hepatitis A**	17 (4.9)	8 (7.1)
**hepatitis B**	57 (16.5)	27 (24.1)
**hepatitis C**	82 (23.7)	34 (30.4)
**hepatitis D**	5 (1.4)	2 (1.8)
**hepatitis E**	2 (0.6)	1 (0.9)

Abbr.: HCC: hepatocellular carcinoma; LT: liver transplantation; NASH: non-alcoholic steatohepatitis.

**Table 2:** HCC expression at initial diagnosis in the entire HCC and the LT cohort.

HCC Characteristics	HCC Cohort (*n* = 346) [*n* (%)]	Patients with LT (*n* = 112) [*n* (%)]
**single lesion**	204 (59.0)	74 (66.1)
**multilocular**	142 (41.0)	38 (33.9)
**inside Milan**	148 (42.8)	74 (66.1)
**inside UCSF**	195 (56.4)	88 (78.6)
**extrahepatic metastases**	28 (8.1)	0
**major vascular invasion**	36 (10.4)	0
**AFP positive**	227 (65.6)	63 (56.3)

Abbr.: AFP: alpha-fetoprotein; HCC: hepatocellular carcinoma; LT: liver transplantation; UCSF: University of California, San Francisco score.

## Data Availability

The clinical datasets supporting the conclusions of this study were derived from the patient files (paper and electronic form). Therefore, restrictions to availability apply due to data protection regulations. Anonymized data are, however, available from the corresponding author on reasonable request and with permission of the University Hospital Schleswig-Holstein and the local review board.
